# Further evidence that item responses on the Kessler Psychological Distress Scale exhibit the characteristic pattern in the general population

**DOI:** 10.1016/j.heliyon.2019.e01387

**Published:** 2019-03-21

**Authors:** Shinichiro Tomitaka, Yohei Kawasaki, Kazuki Ide, Maiko Akutagawa, Yutaka Ono, Toshiaki A. Furukawa

**Affiliations:** a Department of Mental Health, Panasonic Health Center Tokyo, Japan; b Department of Health Promotion and Human Behavior, Kyoto University Graduate School of Medicine/School of Public Health, Kyoto, Japan; c Clinical Research Center, Chiba University Hospital, Chiba, Japan; d Department of Pharmacoepidemiology, Graduate School of Medicine and Public Health, Kyoto University, Kyoto, Japan; e Center for the Promotion of Interdisciplinary Education and Research, Kyoto University, Kyoto, Japan; f Department of Drug Evaluation and Informatics School of Pharmaceutical Sciences, University of Shizuoka, Shizuoka, Japan; g Center for the Development of Cognitive Behavior Therapy Training, Tokyo, Japan

**Keywords:** Public health, Epidemiology, Psychiatry, Clinical psychology

## Abstract

**Background:**

Previous studies suggested that item responses on the 6-item Kessler Psychological Distress Scale (K6) exhibit characteristic distributions among the general population. To confirm the reproducibility of these findings, we conducted a pattern analysis of the K6 item responses using large-scale data from a US representative survey.

**Methods:**

Data were drawn from the 2016, and 2017 National Health Interview Survey in the United States (33,028, and 26,742 individuals, respectively). We analyzed the patterns of item responses for the six items using normal and logarithmic scales and proposed a model of item responses.

**Results:**

The lines for item responses showed the same pattern among the six items, characterized by crossing at a single point between “none” and “a little,” and parallel patterns from “a little” to “all of the time” on a logarithmic scale. The ratio of “some” to “a little,” “most” to “some,” and “most” to “all of the time” were similar across the six items. The model of item responses, which was based on the findings that the decreasing ratios of “some” to “a little,” “most” to “some,” and “all of the time” to “most” were similar across the six items, explained the characteristic patterns of item responses.

**Conclusion:**

These results provide further evidence that item responses on the K6 follow a particular distribution pattern among the general population.

## 1 Introduction

The 6-item Kessler Screening Scale for Psychological Distress (K6) is a self-reported psychological distress scale widely employed in population studies and in primary care [1, 2, 3]. The six items of the K6 (felt nervous, hopeless, restless or fidgety, worthless, sad, and felt that everything was an effort) can be grouped into depressive and anxiety symptoms [4]. The efficacy of the K6 as a screening scale to detect depressive disorders and anxiety disorders has been demonstrated [1]. Although the item response patterns on psychological scales are various in shape, there are few studies that have investigated the reproducible pattern of item responses on psychological scales [5, 6].

**Fig. 1:**
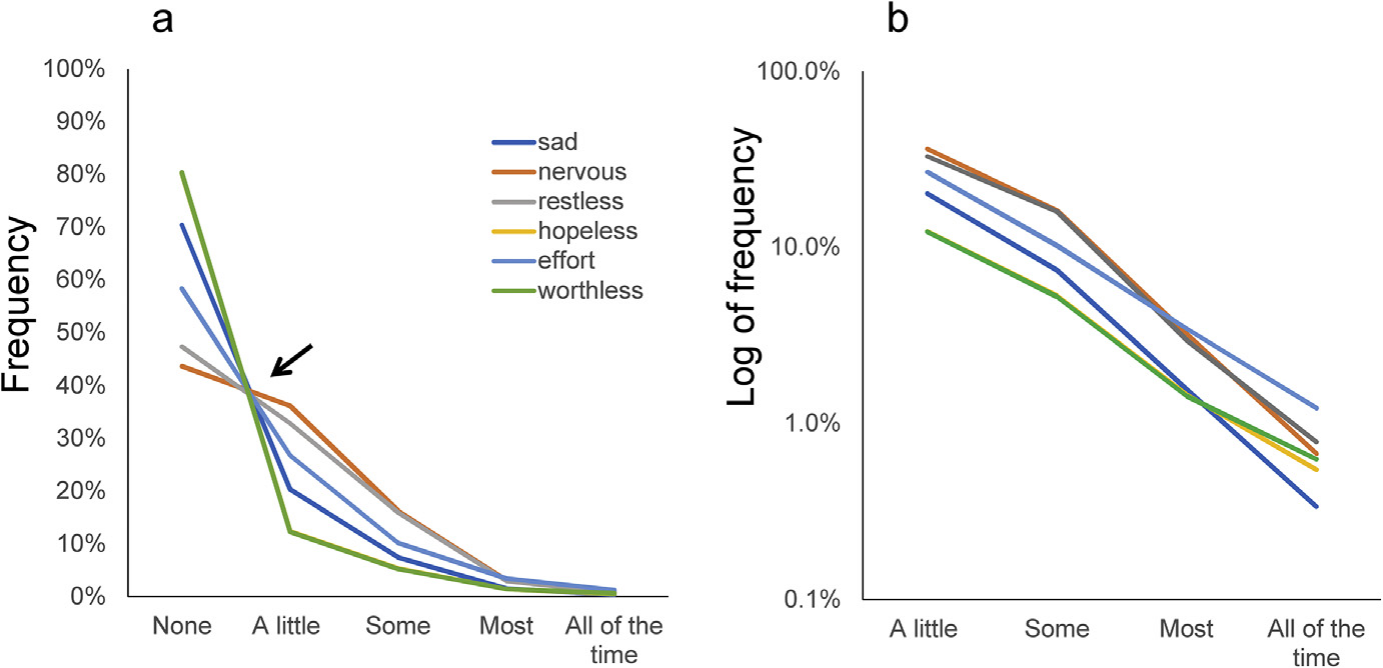
Item responses of the six items using K6 data from the MIDUS. Item responses of the six items of the K6 exhibited a common mathematical pattern on (a) a normal scale and (b) a logarithmic scale. The lines for “hopeless” and “worthless” appear as one line, because two lines are close (a). Image credit: BMC psychiatry at https://doi.org/10.1186/s12888-017-1449-1.

In a previous study, analyzing the K6 data from four subsamples in National Survey of Midlife Development in the United States (MIDUS), we observed that responses to the K6 exhibited a common pattern among the six items (Fig. 1) [7]. The K6 asks individuals to self-report the severity of the six symptoms during the past 30 days with a 5-point scale from “none” to “all of the time” [8]. As shown by the black arrow, the lines for the item responses of the six items appear to cross at a single point between “none” and “a little.” Thereafter, they decrease monotonically from “a little” to “all of the time” (Fig. 1a). When plotted using a logarithmic scale, the lines for all six item responses follow a parallel pattern from “a little” to “all of the time” (Fig. 1b). Of note, in the previous studies, the ratios between two consecutive response options were similar among all six items, except for the response option at the lower end [7, 9].

The reproducibility of such item response patterns has been confirmed for depressive symptom scales. Previous studies have demonstrated that item responses of the Center for Epidemiologic Studies Depression Scale and nine-item Patient Health Questionnaire exhibit the same characteristic patterns among the general population [9, 10, 11]. Moreover, in these previous studies, the ratios between two consecutive response options were similarly observed across all items, except for the option at the lower end, suggesting that the similar ratios between two consecutive response options among all items are strongly related to the characteristic patterns of item response [9, 10, 11].

### 1.1 Aim

To our knowledge, our study is the first to report that all item responses on a psychological distress scale follow a same mathematical pattern. The identified pattern suggests that we may be able to develop an inductive model of item responses on a psychological distress scale. Of note, developing an inductive model using observed data differs from analyzing observed data using statistical models. For example, although item response theory enables us to determine the parameters of item responses on psychological scales, this statistical procedure presupposes item response models, implying that item response theory is distinct from inductively developing a model of observed data without any assumptions [12]. An inductive model of item responses on the K6 is important because it will enable us to better predict how item responses of psychological distress symptoms distribute in the general population. Furthermore, the inductive model determines which statistical procedures to apply. If the item responses follow a non-normal distribution, parametric statistics assuming normal distributions require rethinking [13].

To establish an inductive model of item responses on psychological distress scales, the observed pattern must be confirmed repeatedly using large-scale data. Generally, large-scale data enable researchers to better analyze the pattern of an empirical distribution [14, 15]. In the United States, the K6 has been used as part of the National Health Interview Survey (NHIS) [16]. The NHIS is an annual, cross-sectional survey that provides nationally representative estimates of a range of health status variables among the nonmilitary and noninstitutionalized population, and has been conducted by the National Center for Health Statistics. De-identified data from the NHIS are accessible to researchers worldwide [16]. NHIS data are suitable for use in identifying the aforementioned patterns due to the large sample sizes and limited selection bias. For the present study, therefore, we used data from the NHIS.

Our aim was to demonstrate the characteristics of the item responses on the K6 and determine whether they exhibited the characteristic patterns previously observed. Furthermore, based on the findings of the present study, we sought to propose an inductive model of the item responses on a psychological distress scale.

## 2 Methods

### 2.1 Dataset

The present study data were drawn from the 2016 and 2017 NHIS [16]. The NHIS is a cross-sectional household interview survey of the noninstitutionalized population in all US states and the District of Columbia to make nationally representative estimates of health variables [16]. For each participating family, one adult aged 18 years or older was randomly selected and invited to participate in the interview. The datasets analyzed during the present study are available in the National Health Interview Survey repository, https://www.cdc.gov/nchs/nhis/data-questionnaires-documentation.htm.

### 2.2 Ethics statement

The present study used de-identified data available to the public. Since the ethics committee of the Panasonic Health Center does not consider de-identified public data analysis as human subjects research, the committee ruled that institutional review board approval was not necessary for the present project.

### 2.3 Measures

The K6 includes six items related to the degree with which participants have felt sad, nervous, restless, hopeless, that everything was an effort, and worthless over the previous 30 days. Each item is self-rated with 5-point response options from 0 = “none of the time” to 4 = “all of the time,” yielding a total item score of 0–24. One of the K6 items used in the NHIS survey is worded as follows: “How much of the time did you feel so sad that nothing could cheer you up?” This wording is still used by the NHIS today and a little different from the wording used in the K6 today, which is as follows: “How often did you feel so depressed that nothing could cheer you up?”

### 2.4 Analysis

In the previous studies, the ratios between two consecutive response options have been reported to be similar among all items, except for the option at the lower end [7, 9]. Thus, the ratios of “some” to “a little,” “most” to “some,” and “all of the time” to “most” were calculated for all six items. To show the degree of similarity in these ratios, relative standard deviation, or coefficient of variation, for these ratios was calculated. Next, we graphically analyzed the patterns of item responses for the six items using normal and logarithmic scales. In general, graphical analysis is required for pattern analysis of complex models because it enables us to easily detect a pattern of data. If the exact same data were only presented in a numerical table, we would have overlooked its complicated pattern. By plotting all item response rates together on the same graph, we sought to identify a common pattern of responses among all items. Finally, as the line graphs for item responses showed the same mathematical pattern among the six items, we attempted to formulate a mathematical model that could explain it. Based on the results that the ratios of “some” to “a little,” “most” to “some,” and “all of the time” to “most” were similar among the six items, an inductive model of the item responses on the K6 was proposed. JMP software, Version 12 for Windows (SAS Institute, Inc., Cary, NC, USA) was used to conduct the analysis.

## 3 Results

### 3.1 Item response analysis

The 2016 and 2017 NHIS samples included 33,028, and 26,742 respondents, respectively, and the final response rates were 54.3% and 53.0%, respectively. The demographic characteristics of the NHIS participants are reported in detail elsewhere [16].

Data from participants who did not report the response options for all items were excluded from the item response analysis. Thus, the final sample for the analysis included 31,889 and 25,767 individuals from the 2016 and 2017 NHIS, respectively. The excluded sample comprised 1,189 (3.6%) and 975 (3.6%) persons, respectively.

Table 1 depicts the item response rates for all six items in the 2016 and 2017 NHIS. Item response rates exhibited a similar pattern among the six items — the highest response rate being for “none,” a decreasing response rate as item scores increased, and the lowest rate observed for “all of the time” (Table 1). The relative standard deviation for the ratios of “some” to “a little,” “most” to “some,” and “all of the time” to “most” for the 2016 and 2017 NHIS samples were 0.14 and 0.13, 0.14 and 0.15, and 0.22 and 0.23, respectively, indicating that the ratios of “some” to “a little,” “most” to “some,” and “all of the time” to “most” were similar among the six items. The exception was that the rate of “all of the time” to “most” for “sad” was considerably lower than for the other K6 items. The average ratio of “most” to “some” (0.28 and 0.27) was lower than those of “some” to “a little” (0.80 and 0.78), and “all of the time” to “most” (0.73, and 0.74) for the 2016 and 2017 NHIS samples, respectively (Table 1).

**Table 1:** Item responses of the 2016 and 2017 NHIS samples.

Item	Item response, n (%)					Rate of “some” to “a little”	Rate of “most” to “some”	Rate of “all” to “most”
	None	A little	Some	Most	All of the time			
**The 2016 National Health Interview Survey**								
Sad	23 697 (74)	4412 (14)	2700 (8)	747 (2)	333 (1)	0.61	0.28	0.45
Nervous	19 863 (62)	5946 (19)	4332 (14)	961 (3)	787 (2)	0.73	0.22	0.82
Restless	20 158 (63)	5161 (16)	4364 (14)	1087 (4)	1019 (3)	0.85	0.27	0.86
Hopeless	27 486 (86)	2001 (6)	1661 (5)	451 (1)	290 (1)	0.83	0.27	0.64
Effort	22 785 (71)	3698 (12)	3319 (10)	1114 (3)	973 (3)	0.90	0.34	0.87
Worthless	28 430 (89)	1444 (5)	1290 (4)	411 (1)	314 (1)	0.89	0.32	0.76
Average	23 737 (74)	3777 (12)	2944 (9)	812 (3)	619 (2)	0.80 ± 0.11	0.28 ± 0.04	0.73 ± 0.16
**The 2017 National Health Interview Survey**								
Sad	19 208 (75)	3558 (14)	2207 (9)	554 (2)	240 (1)	0.62	0.27	0.43
Nervous	15 857 (62)	4816 (19)	3649 (14)	843 (3)	602 (2)	0.76	0.23	0.71
Restless	16 085 (62)	4203 (16)	3625 (14)	983 (4)	871 (3)	0.86	0.27	0.89
Hopeless	22 155 (86)	1744 (7)	1272 (5)	350 (1)	246 (1)	0.73	0.28	0.70
Effort	18 071 (70)	3108 (12)	2805 (11)	948 (4)	835 (3)	0.90	0.34	0.88
Worthless	22 979 (89)	1243 (5)	1025 (4)	287 (1)	233 (1)	0.82	0.28	0.81
Average	19 059 (74)	3112 (12)	2431 (9)	661 (3)	505 (2)	0.78 ± 0.10	0.27 ± 0.04	0.74 ± 0.17

Average rate data are presented as the mean plus or minus one standard deviation.

### 3.2 Graphical analysis

**Fig. 2:**
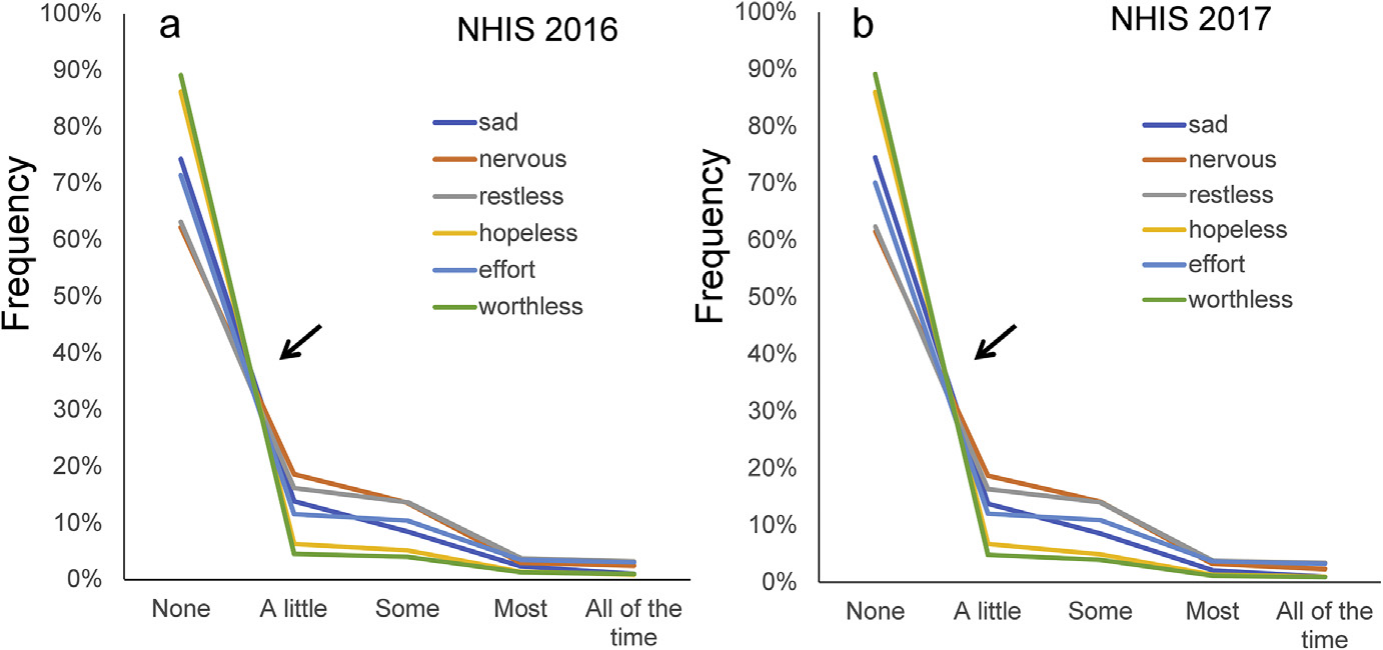
K6 item responses on normal scales. (a) NHIS 2016 sample, and (b) NHIS 2017 sample on normal scales. As indicated by the arrows, the lines for the six items cross at a single point between “none” and “a little,” whereas the lines from “a little” to “all of the time” decrease regularly.

To analyze the pattern of item responses, we performed a graphical analysis, with all six item response rates plotted together on the same graph. The item responses for the 2016 and 2017 NHIS are shown in Fig. 2a and b. The item responses of the six items demonstrated a common pattern of different types of distributions with a boundary at the rating of “a little.” As indicated by the arrows in Fig. 2a and b, the lines for the six items appeared to cross at a single point between “none” and “a little,” thereafter they converged from “a little” to “all of the time,” consistent with the patterns reported in previous studies (Fig. 1a) [7, 9].

**Fig. 3:**
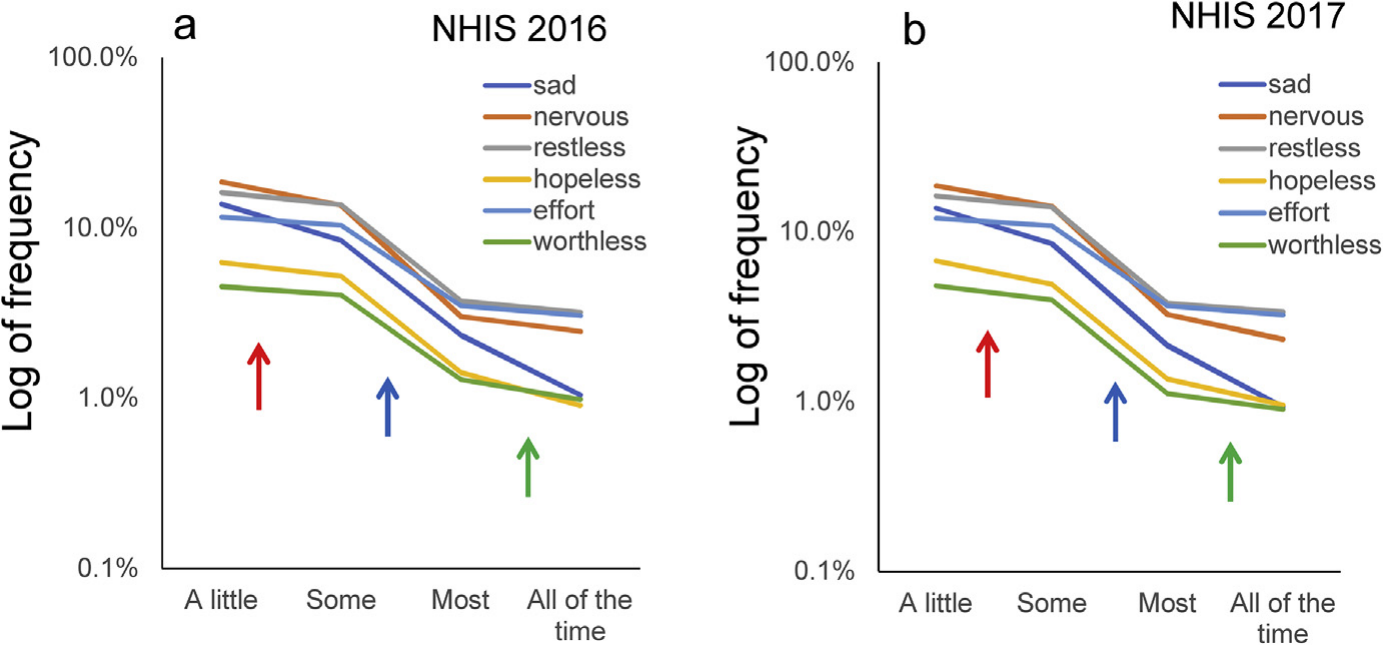
K6 item responses on logarithmic scales. (a) NHIS 2016 sample, and (b) NHIS 2017 sample on logarithmic scales. The gradients of the linear patterns of the K6 item responses are gentle between “a little” and “some” (red arrows), steep between “some” and “most” (blue arrows), and gentle again between “most” and “all of the time” (green arrows).

Using a logarithmic scale, the lines of the K6 items generally decreased in parallel from “a little” to “all of the time” (Fig. 3a and b). The exception was that the line for “sad” was less horizontal than the other K6 items between that for “most” to “all of the time,” consistent with the result that the rate of “all of the time” to “most” for “sad” was considerably lower than that for the other K6 items. As shown in Fig. 3a and b, the gradients of the linear patterns of the K6 item responses differed at the level of item response. The gradients of the linear patterns of the K6 item responses were gentle between “a little” and “some” (red arrows), steep between “some” and “most” (blue arrows), and gentle again between “most” and “all” (green arrows). These observations are consistent with the average decreasing ratios of “some” to “a little” and “all of the time” to “most,” being larger compared to that of “most” to “some” (Table 1).

### 3.3 Inductive model of item responses

**Fig. 4:**
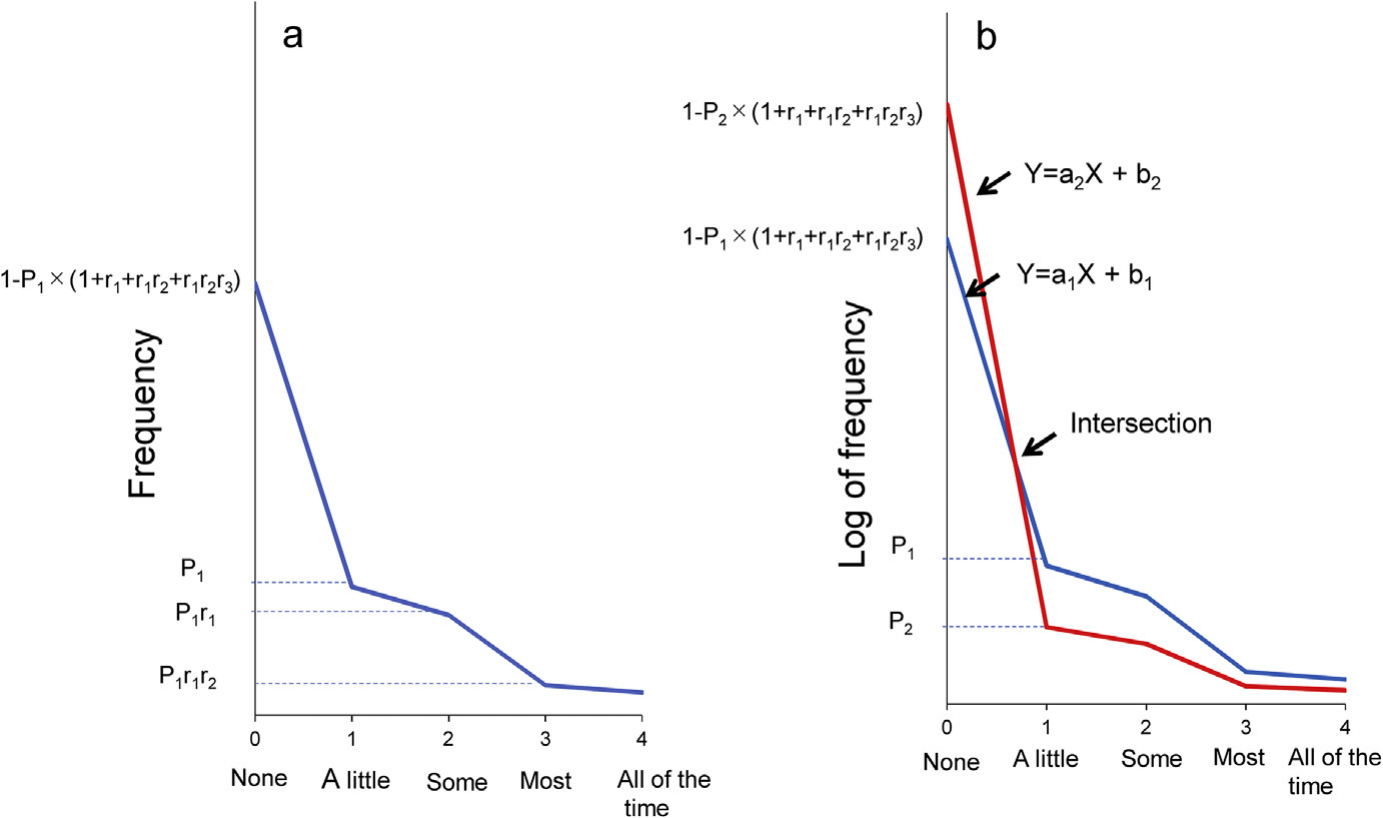
Inductive model and crossing at a single point between “none” and “a little”. (a) This mathematical model is based on the results that the decreasing ratios of “some” to “a little,” “most” to “some,” and “all of the time” to “most” were constant among the six items. (b) A model of the intersection of two lines of item responses.

Based on the results that the decreasing ratios of “some” to “a little,” “most” to “some,” and “all of the time” to “most” were similar across the six items, the inductive model of the item responses on the K6 was proposed (Fig. 4). As shown in Fig. 4a, when the probability of “a little” is presented as P_1_ and the ratio of “some” to “a little,” “most” to “some,” and “all of the time” to “most” are presented as three constants, r_1_, r_2_, and r_3_, the probabilities of “some,” “most,” “all,” and “none” are expressed as P_1_r_1_, P_1_r_1_r_2_, P_1_r_1_r_2_r_3_, 1 − P_1_ × (1 + r_1_ + r_1_r_2_ + r_1_r_2_r_3_), respectively. The scores of “none,” “a little,” “some,” “most,” and “all of the time” are expressed as 0, 1, 2, 3, and 4, respectively. The present mathematical model is more generalized than the previous model, which did not predict the decreasing ratios of item responses differing according to the level of item response [9].

In this study, the lines for the six items crossed at a single point between “none” and “a little.” The present model may explain the phenomenon of lines crossing at a single point between “none” and “a little.” As shown in Fig. 4a, the line from “none” to “a little” is expressed as:

Y = a_1_X + b_1_,

where a_1_ is the gradient and b_1_ is the intercept of the line.

Then, a_1_ and b_1_ can be expressed as follows:

a_1_ = P_1_ × (r_1_r_2_r_3_ + r_1_r_2_ + r_1_ +2) − 1,

and

b_1_ = 1-P_1_ × (r_1_r_2_r_3_ + r_1_r_2_ + r_1_ +1).

As shown in Fig. 4b, the two lines between “none” and “a little” are expressed as follows:

Line 1: Y = a_1_X + b_1_,

and

Line 2: Y = a_2_X + b_2_,

where,

a_1_ = P_1_ × (r_1_r_2_r_3_ + r_1_r_2_ + r_1_ + 2) − 1,

b_1_ = 1 − P_1_ × (r_1_r_2_r_3_ + r_1_r_2_ + r_1_ + 1),

a_2_ = P2 × (r_1_r_2_r_3_ + r_1_r_2_ + r_1_ + 2) − 1,

and

b_2_ = 1 − P_2_ × (r_1_r_2_r_3_ + r_1_r_2_ + r_1_ + 1).

The point where the two lines cross is then expressed as follows:

X = (r_1_r_2_r_3_ + r_1_r_2_ + r_1_ + 1) / (r_1_r_2_r_3_ + r_1_r_2_ + r_1_ + 2),

and

Y = 1 / (r_1_r_2_r_3_ + r_1_r_2_ + r_1_ + 2).

The intersection is expressed by r_1_, r_2_, and r_3_ only. Therefore, if the decreasing ratios of “some” to “a little,” “most” to “some,” and “all of the time” to “most” are constant among the six items, all the lines must cross a single point between “none” and “a little” (Figs. 2 and 4b).

**Fig. 5:**
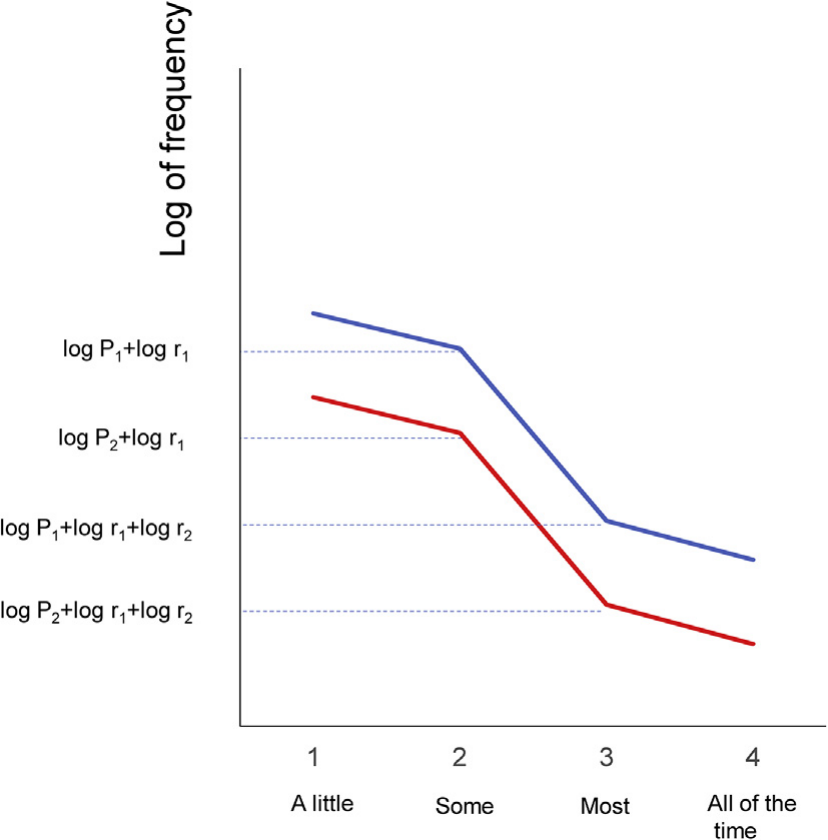
Inductive model and a parallel pattern between “some” and “most”. A model of the parallel pattern between “some” and “most” on a logarithmic scale.

The finding that the lines for the six items exhibit a parallel pattern from “some” to “all of the time” can also be explained by the present model. Fig. 5 illustrates the distributions of the six items between “some” and “most” when plotted on a logarithmic scale. Using a logarithmic scale, the probabilities of “some” and “most” for the blue line are expressed as logP_1_ + logr_1_, and logP_1_ + logr_1_ + logr_2_. Similarly, the probabilities of “some” and “most” for the red line are expressed as logP_2_ + logr_1_, and logP_2_ + logr_1_ + logr_2_ (Fig. 5). Consequently, the red and blue lines have the same slope (logr_2_) between “some” and “most” on a logarithmic scale, indicating that the two lines exhibit a parallel pattern from “some” to “most” on a logarithmic scale. In the same manner, since the decreasing ratios of “some” to “a little,” “most” to “some,” and “all of the time” to “most” are similar across the six items, the lines for the six items exhibit a parallel pattern from “a little” to “all of the time” on a logarithmic scale.

## 4 Discussion

The present study aimed to investigate the patterns of item responses on the K6 using the NHIS data. The main findings were as follows: (1) the decreasing ratios of “some” to “a little,” “most” to “some,” and “all of the time” to “most” were similar across the six items of the K6; (2) the lines for item responses demonstrated the same pattern among the six items, characterized by crossing at a single point between “none” and “a little,” and parallel patterns from “a little” to “all of the time” on a logarithmic scale; (3) the inductive model of item responses explained the characteristic patterns of item responses; and (4) the average ratio of “most” to “some” was lower than those of “some” to “a little” and “all of the time” to “most.” These findings provide further evidence that item responses on the K6 follow a characteristic pattern in the general population.

Although the inductive model has revealed that the characteristic patterns of item response are caused by the similar ratios of “some” to “a little,” “most” to “some,” and “all of the time” to “most” across the six items of the K6, it remains unclear how these ratios were similar across the six items. The mechanism of such similar ratios can be speculated on from respondent's viewpoint. When respondents fill out psychological distress scales, they first assess whether the given symptoms are present. If each respondent does not notice the symptom, then it is categorized as “absence” (i.e., it is rated as “none” on the K6). Next, if each respondent notices the symptom, its severity is categorized according to the remaining degree-adverb options (i.e., it occurs “a little,” “some,” “most,” or “all of the time”). These processes imply that “absence” covers the under-threshold range while the degree-adverb options cover the above-threshold range. Thus, if each of the degree-adverb options covers the fixed proportion of the above-threshold range, the characteristic pattern of item responses will emerge [17]. Further studies are needed to determine how each of the degree-adverb options covers the fixed proportion of the above-threshold range.

In this study, the average ratios of “some” to “a little,” “most” to “some,” and “all of the time” to “most” varied according to the level of item responses (Table 1). The average ratios of “some” to “a little” and “all of the time” to “most” were two and half times larger than that of “most” to “some,” resulting in the apparent fluctuations of log-normal scales (Fig. 3a and b). In a previous analysis of the K6 data in the MIDUS survey, the average decreasing rates of “some” to “a little,” “most” to “some,” and “all of the time” to “most” were 0.43, 0.25, and 0.31, respectively (Fig. 1a) [7]. Furthermore, according to our analysis of K6 data from nearly 40,000 respondents participating in the 2014 National Survey on Drug Use and Health, the average decreasing rate of “some” to “a little,” “most” to “some,” and “all of the time” to “most” were 0.53, 0.30, and 0.54, respectively [18]. These results suggest that for the K6 scale, there is a tendency for the decreasing rate of “most” to “some” to be lower than the rates of “some” to “a little” and “all of the time” to “most” in the general population.

Our results show that the decreasing ratio of “all of the time” to “most” for “sad” was considerably lower than for the other K6 items. This finding is consistent with the previous results from the MIDUS (Fig. 1). Although the reason for this is unclear, these results suggest that the item “sad” could exhibit slight different pattern of item responses from the other K6 items. In that case, it is necessary to modify the inductive model of item responses according to the items. Further studies are necessary to confirm whether the item response pattern of “sad” differs from those of other items.

The present study has a number of limitations. We evaluated the similarities among distributions by calculating the ratios of “some” to “a little,” “most” to “some,” and “all of the time” to “most” for all six items. Moreover, we used graphical analysis to demonstrate the similarities among distributions. However, one major limitation of these methods is the lack of unified criteria to interpret the results. Thus, even after obtaining the results of these analyses, we were unable to determine the degree of similarity or difference of the factors in the complex pattern using unified criteria; this includes comment on significance. Further research is necessary to develop unified criteria for the interpretation of these results.

Conversely, our study has methodological advantages. The graphical analysis enabled a complex pattern of item responses to be identified. In general, graphical analysis is essential for exploratory data analysis of complex models [19].Furthermore, the data were taken from the NHIS, thus ensuring a large sample size with limited selection bias. In addition, confirmation using samples from two different years ensured the reproducibility of the findings.

In psychological studies, the normal distribution is the widely used in statistical theory and applications [8]. However, to our knowledge, there is little evidence that item scores on psychological distress scales follow a normal distribution in the general population [5, 6]. Our results using an inductive model suggest that statistical procedures assuming a normal distribution (e.g., Student's t-test, Pearson's correlation, and factor analysis) will require reconsideration when used to analyze item responses on psychological distress scales.

Recent studies using clinimetric analysis reported the clinical utility of using item response theory models to rate the severity of a symptom [20, 21, 22]. Item response models are based on the assumption of a unidimensional latent trait. However, there is little evidence that all item responses have some common characteristics. The common characteristic pattern of item responses among all psychological distress symptoms leads us to speculate that these symptoms share a common process for the manifestations.

## 5 Conclusions

The results of this study suggest that the responses to the psychological distress items of the K6 follow the same mathematical patterns in the general US populations. The present study provides further evidence of how item response on psychological distress scales is distributed in the general population. Further studies should focus on the mechanism that governs the mathematical patterns of item responses on psychological distress scales in the general population.

## Declarations

### Author contribution statement

Shinichiro Tomitaka: Conceived and designed the experiments; Performed the experiments; Analyzed and interpreted the data; Wrote the paper.

Yohei Kawasaki, Kazuki Ide, Maiko Akutagawa, Yutaka Ono, Toshiaki A. Furukawa: Analyzed and interpreted the data; Wrote the paper.

### Funding statement

This work was supported by a research grant from the JSPS KAKENHI (grant number 18K03145).

### Competing interest statement

The authors declare no conflict of interest.

### Additional information

No additional information is available for this paper.
